# “TREXIT 2020”: why the time to abandon transrectal prostate biopsy starts now

**DOI:** 10.1038/s41391-020-0204-8

**Published:** 2020-01-13

**Authors:** Jeremy Grummet, Michael A. Gorin, Rick Popert, Tim O’Brien, Alastair D. Lamb, Boris Hadaschik, Jan Philipp Radtke, Florian Wagenlehner, Eduard Baco, Caroline M. Moore, Mark Emberton, Arvin K. George, John W. Davis, Richard J. Szabo, Roger Buckley, Andrew Loblaw, Matthew Allaway, Christof Kastner, Erik Briers, Peter L. Royce, Mark Frydenberg, Declan G. Murphy, Henry H. Woo

**Affiliations:** 1 Department of Surgery, Central Clinical School, Monash University, Melbourne, VIC Australia; 2 Department of Urology, The James Buchanan Brady Urological Institute, Johns Hopkins University School of Medicine, Baltimore, MD USA; 3 Guy’s and St Thomas’ Hospital, London, UK; 4 Oxford University, Oxford, UK; 5 Essen University Hospital, Essen, Germany; 6 Justus-Liebig University, Gießen, Germany; 7 Oslo University Hospital, Oslo, Norway; 8 University College London, London, UK; 9 University of Michigan, Ann Arbor, MI USA; 10 MD Anderson Cancer Center, Houston, TX USA; 11 Southern California Kaiser Permanente, Los Angeles, CA USA; 12 North York General Hospital, North York, ON Canada; 13 Odette Cancer Centre, Sunnybrook Health Sciences Centre, University of Toronto, Toronto, ON Canada; 14 Urology Associates, Baltimore, MD USA; 15 Addenbrookes Hospital, Cambridge University, Cambridge, UK; 16 European Cancer Patient Coalition, Brussels, Belgium; 17 Monash University, Clayton, VIC Australia; 18 Peter MacCallum Cancer Centre, University of Melbourne, Melbourne, VIC Australia; 19 Sydney Adventist Hospital, University of Sydney, Sydney, NSW Australia

**Keywords:** Prostate cancer, Prostate cancer

In 1847, 20 years before germ theory was popularised by Louis Pasteur, the Hungarian physician Ignaz Semmelweis famously reduced maternal mortality from post-partum sepsis from 16 to 1% simply by encouraging hand hygiene among his peers [1]. Despite the evidence, many physicians of the day were offended by the assertion that they themselves may be the cause of patient deaths and rejected Semmelweis’s life-saving advice. Aged just 47, he suffered a nervous breakdown, was committed to an asylum and died within 2 weeks, ironically and tragically, from a gangrenous wound.

Like Semmelweis, urologists today have the opportunity to nearly eliminate infections we cause by performing transrectal (TR) prostate biopsy and switch instead to the clean transperineal (TP) approach—a process our co-authors at Guy’s Hospital in London, UK, have opportunistically dubbed “TRexit” [2, 3].

Despite the recent advances in prostate cancer imaging with MRI [4] and PSMA PET [5], a biopsy is still required to establish a diagnosis of prostate cancer. The vast majority of prostate biopsies are still performed using the TR approach—over 2 million per year in Europe and North America alone [6]. However, in recent years TP biopsy has gained increasing favour due to its avoidance of rectal flora [7].

By passing the biopsy trocar from dirty to clean, TR biopsy breaks the fundamental surgical principle of sterile technique. The procedure is thus plagued by the potential for inoculation of a large dose of rectal bacteria into the bloodstream. Despite the use of standard antibiotic prophylaxis, typically a fluoroquinolone, due to the emergence of multi-drug resistant bacteria, post-TR biopsy infection is increasing [6, 8] and was recently reported to be alarmingly high at 10% [9]. TR biopsy sepsis can also be life-threatening. Its mortality rate is 0.13% of TR biopsies in Taiwan [10], and was calculated at an additional ten deaths per year in Norway (population 5 million) [9].

To combat this problem, clinicians have resorted to escalating the type of prophylactic antibiotic employed [11], with some suggesting the use of carbapenems [12, 13]. Whilst this may reduce the sepsis rate [14], it is in direct opposition to the advice from the US Center for Disease Control [15]. Both the US Food and Drug Administration [16] and the European Medicines Agency [17] have recently issued strong warnings recommending against the use of fluoroquinolones also.

Not only is there the obvious human cost of suffering from TR-biopsy related infections, but there is also the financial burden. Analysis of an Australian government Department of Health database revealed that the mean cost per admission was US$6844 [18]. This did not take into account loss of productivity of patients or carers. More recently in the United States, the estimated cost of post-biopsy sepsis was between US$8672 and US$19,100 per patient [19].

TP biopsy, on the other hand, avoids rectal flora altogether. Whilst there are no RCTs directly comparing TR and TP biopsy infection, the differences in infection rates are stark, with sepsis from TP biopsy approaching zero. This is regardless of whether just a single dose of first-generation cephalosporin is used [20], or antibiotic prophylaxis is omitted altogether [21]. This lack of sepsis has been shown in numerous studies [22–25], including a series of 1194 consecutive TP biopsies performed across five centres in Melbourne, Australia, in which the re-admission rate for infection was zero [26]. TP biopsy became standard practice by these authors in 2012.

Regarding detection of significant cancer, TP biopsy is at least equivalent to TR biopsy, with some evidence that TP biopsy offers superior detection of anterior tumours [27].

Some authors have cited the increased rate of acute urinary retention (AUR) with the TP approach as an argument against its use. However, the largest series of 1287 consecutive biopsies at North York General Hospital in Toronto, Canada, reported the rate at just 1.6% [23]. Conversely, AUR was as high as 24% in the PICTURE study [28]. Erectile dysfunction (ED) was also noted in this study. Notably, this cohort received a median of 49 cores at 5 mm intervals, taken as a systematic mapping biopsy. Most TP systematic biopsies recommend less than half this number [18–24] of cores. Whilst patients should be advised of the risks of AUR and transient ED in TP biopsy (as they should in TR biopsy also), neither of these complications are life-threatening.

Until recently, the greatest deterrent to widespread uptake of TP biopsy has been logistical. Whereas TR biopsy can readily be performed in the office under local anaesthesia (LA), TP biopsy has historically required use of a grid-stepper unit so that general anaesthesia (GA) has been used for men to tolerate the multiple needle passes through the perineum. Whilst TP biopsy under LA has been successfully performed using a grid-stepper unit [29], a new and parallel skin puncture is required for every biopsy taken, requiring a broad area of LA coverage.

The development of freehand techniques for performing TP biopsy, which employ two common access cannulae through the perineal skin, has made it possible for this procedure to now be performed far more readily under LA. In the largest study to date, the 1287 aforementioned Toronto patients underwent a systematic TP biopsy under LA (LATP biopsy) using one such freehand technique [23]. A minimum of ten cores were taken. Patients tolerated the procedure well and none were admitted for infection. A challenge with the method described by this group is the use of a simple common access cannula, which is not coupled to the ultrasound probe. While the authors achieved mastery of this technique within a 6-week learning curve, the needle not being maintained in line with the ultrasound probe makes it difficult for the user to track the location of the needle relative to the probe.

This issue has since been addressed with the introduction of the PrecisionPoint Transperineal Access System™ (Perineologic, Cumberland, MD, USA), which attaches to the ultrasound probe and maintains a common access cannula in line with the probe. This simple device has revolutionized freehand MRI-targeted and systematic LATP biopsy and its successful use has been described by groups in the United States and UK [21, 30–32]. Notably, LATP biopsy can be achieved using any ultrasound probe currently used for TR biopsy, as long as the prostate can be viewed in the sagittal plane and an access system attached to the probe—a technique first described by the group in Oxford, UK [33, 34].

The major barriers to implementation of in-office TP prostate biopsy, namely the increased capital costs for linear array brachytherapy probes, grid-stepper units and the need for GA, have therefore now been removed.

Recognising its improved patient safety, we believe LATP biopsy should now become standard of care. As such, healthcare payers and policymakers should facilitate adoption of this practice. However, we must first ensure appropriate training and equipment are made available to the urologic community.

The TRexit initiative, run by the South East London Cancer Alliance that comprises six hospitals serving 1.5 million people, is a project doing just this. Through provision of training and resources, the TRexit initiative successfully ceased all TR biopsies and converted to LATP biopsy in March 2019, (days before the UK government had planned, but failed, to deliver Brexit) [2]. The TRexit initiative aims to have TR biopsy replaced right across the UK. TRexit has also occurred in Norway due to the recent widely publicized post-TR biopsy patient death and local sepsis rate of 10%, compared with the zero rate of post-biopsy infection at Oslo University Hospital when TP biopsy was introduced [9].

In conclusion, we ask that our colleagues do not bestow the same fate suffered by Semmelweis on those who champion TP biopsy. The mechanism underlying TR-biopsy-related sepsis is clear and can be readily avoided using the TP approach, which is now also feasible under LA. We believe a well-planned global TRexit, with a phase-out period of TR biopsy led by centres experienced in TP biopsy, should be instigated in 2020, aiming for completion by the end of 2022.
